# Universal health coverage in Pakistan: is the health system geared up to take on the challenge?

**DOI:** 10.1186/s12992-023-00904-1

**Published:** 2023-01-12

**Authors:** Babar Tasneem Shaikh, Nabeela Ali

**Affiliations:** JSI Research & Training Institute Inc, Mezzanine Floor, Grand Hotel, Street 1, MPCHS, E-11/1, Islamabad, 44000 Pakistan

**Keywords:** Health system, Health service delivery, Out of pocket expenditure, Population coverage, Universal health coverage, Pakistan

## Abstract

**Background:**

There is a strong and wide consensus that Pakistan must pursue universal health coverage (UHC) attainment as the driving force for achieving sustainable development goals by 2030. Nevertheless, several institutional and socioeconomic challenges may hinder the progress toward UHC.

**Main body:**

It is important that the health system of Pakistan must be transformed to strengthen all three dimensions of UHC i.e. maximizing the population covered, increasing the range of services offered, and reducing the cost-sharing. To make UHC dream a reality in Pakistan, there are some pre-requisites to meet upfront: a) budgetary allocation for health as percentage of GDP must be increased; b) health system’s readiness especially in the public sector ought to improve in terms of human resource and availability of essential services; c) safety nets for health must continue regardless of the change in the political regimes; d) decrease the reliance on donors’ funding; and e) accountability to be ensured across the board for service providers, managers, administrators and policymakers in the health system.

**Conclusion:**

COVID-19 pandemic has revealed some major gaps in the health system’s capacity to deliver equitable healthcare, which is a cornerstone to achieving the UHC agenda. The priority-setting process will need to be aligned with the SDGs to ensure that the agenda for action towards 2030 is comprehensively addressed and successfully accomplished preferably before, but hopefully not beyond the targeted dates.

## Background

Universal Health Coverage (UHC) is a principle that aims to ensure that all people, particularly those in need, have access to essential health services when and where they need, without any financial hardship. The sustainable development goal 3.8, adopted in 2015 by the United Nations reflects UHC [1]. Although the use of UHC term was introduced in 2005 by the WHO member states [2], the vision and spirit of UHC was first substantiated by the WHO founding constitution that stipulated health as a universal right, and recognized in its constitution in 1948 that “the enjoyment of the highest attainable standard of health is one of the fundamental rights of every human being without distinction of race, religion and political belief, economic and social condition” is one of its cardinal principles [3]. This UHC concept was globally reaffirmed in 1978 through the Declaration of Alma-Ata International Conference on Primary Health Care, where PHC was identified as the key to the achievement of Health for All by 2000 [4].

World Health Organization emphasized maximum population coverage, health service coverage, and financial protection, as three dimensions of UHC [5]. The first dimension is relatively simpler to understand: what proportion of the people in the catchment of a health facility are covered for health services, and how many are left out. Reasons can be multiple and diverse, but ought to be minimized to ensure that the maximum population benefits from the essential health services available at the health facility. The second dimension is about the range of essential health services made available to the people e.g. immunization, family planning, antenatal care, delivery by skilled birth attendant, treatment of common ailments, and services for HIV/AIDS, tuberculosis and malaria, etc. Third dimension is a bit complex and explains the expenditure incurred on health services. Besides the state financing or pooling of funds, the out of pocket expenditure determines the cost sharing by the patient, which is supposed to be minimal or zero at the point of service delivery. If the gap is large, the incidence of catastrophic expenditure and probability of impoverishment of the families is high due to purchasing of health. Hence, the third dimension of UHC calls for financial risk protection [2, 6].

The current situation of UHC in Pakistan is not very encouraging as compared to the neighboring countries as shown in the Table 1 below:

**Table 1:** Some indicators depicting UHC service coverage index in the region

Indicators	Pakistan	India	Bangladesh	Iran
Out of pocket expenditure on health	54	55	73	39
Child immunization (DPT3)	≥80	≥80	≥80	≥80
Family planning demand satisfied	50	73	73	76
Antenatal care 4+ visits	52	51	37	≥80
International Health Regulations Core Capacity index	49	78	67	80
UHC index	45	61	51	77

Source: WHO & World Bank. Tracking Universal Health Coverage: 2021 global monitoring report. Geneva: 2021

### UHC from dream to reality in Pakistan

Pakistan’s health system has imprinted structural and non-structural barriers, impeding and denying access to health services for the poor and marginalized groups, including women who continue to be disproportionately affected due to poor quality of care and lack of availability of basic essential commodities and services [6]. Country must develop a more gender and pro-equity focussed urban health strategy and costed action plans aligned with the national and provincial health plans, through the expansion of a female health workforce, in combination with institutional policies to support improved service availability [7]. There is a strong and wide consensus that Pakistan must pursue UHC attainment as the driving force for achieving the SDGs. Nevertheless, several institutional and socioeconomic challenges may hinder the progress towards UHC, and the necessary actions need to be taken to overcome these factual barriers that are delineated below:

- **Sub-optimal health indicators:** With the GDP allocation consistently of less than 1% for health [8], what miracles one would expect with regard to the health status of population. Although Pakistan’s health indicators have improved in the last decade owing to better health awareness, improved access to health services and women’s literacy; these remain behind as compared to those of other neighboring countries in south Asia and in the Eastern Mediterranean region [9]. The Pakistan Demographic & Health Survey of 2017-18 revealed improvements in immunization coverage, antenatal care, deliveries assisted by skilled birth attendants, under 5 and neonatal mortality. This could be mainly attributed to the fact that most of the primary healthcare seeking is in the private sector and that too out of pocket. However, since the private sector especially general physicians by and large do not provide family planning services and people have to rely on the government’s family welfare clinics, the contraceptive prevalence rate remained unchanged, rather declined in a few provinces [10]. Maternal mortality ratio has also improved, yet one of the highest in the region [11]. Although the country is well poised to achieve the SDGs, nevertheless it requires huge efforts at all levels of the health system. COVID-19 pandemic has further contributed to worsening of the health indicators particularly those related to maternal, newborn and child health, because the outpatient services were suspended by the provincial governments and the issue of accessibility became even more crucial, till the Supreme Court of Pakistan had to intervene to order the resumption of outpatient services of all hospitals [12]. Data from DHIS showed downward trends of antenatal care seeking, institutional deliveries, family planning, consultations for newborns and care seeking for children under 5 years for pneumonia and diarrhea, and routine immunization [13]. The detrimental effects of COVID-19 and the containment strategies and restrictions adopted by the government of Pakistan may reflect relatively higher rates of maternal, newborn and child health indicators in the next PDHS, resulting in stalling the progress towards SDG3 and UHC. The country would need to not only recover lost ground but quickly accelerate in a now shortened amount of time.
- **Weak health services delivery:** The high mortality is predominantly observed among the vulnerable rural population groups, to which the state provisioned health resources are the least available. Although the country has one of the best health system infrastructure networks, the operationalization of the district health system suffers due to a shortage of a qualified health workforce, especially nurses, midwives and pharmacists and the skill mix is skewed towards doctors, who prefer to run their for-profit private practice [14, 15]. In Pakistan, the doctor-population ratio, the nurse-population ratio and the hospital beds to population ratio are all below the standards proposed by the WHO [16]. Even for the provision of essential and basic quality health services, minimal human resource is required with refreshed knowledge, an optimum set of skills and a few core competencies. The overall dearth of healthcare providers, and the skewed deployment of the existing workforce in big urban hospitals, has incapacitated the healthcare system to provide even the essential health services to the population it is supposed to serve with the tax-payers’ money [17]. UHC index (which is computed as the geometric mean of 14 tracer indicators of health service coverage) for Pakistan has risen from 23 in 2000 to 45 in 2017, on a scale of 100 [18]. Similarly, a strong national public health system in Pakistan is needed to assess the magnitude of public health risks, share real-time information, and implement public health control measures in a concerted and systematic demeanor [19]. There is still a long way to go in terms of improving access, utilization and eventually the desired health outcomes.
- **High out-of-pocket (OOP) spending on healthcare**: The private sector in Pakistan is the major preferred health service provider of all levels of health care, owing to the abysmal state of public sector health facilities [20]. The quality and responsiveness are also relatively better; however, the OOP expenditure remains the biggest challenge, while seeking care without any health insurance or social protection for health, effectively limiting access to essential lifesaving health services. When the OOP is almost 56-60% of the total expenditure on health [21], it is likely to lead to the impoverishment of the already poor families. Health shocks otherwise have been the foremost cause of pushing people into the poverty trap in Pakistan [22]. Government of Pakistan has launched the social health protection initiative *‘Sehat Sahulat (health convenience) Program’* which is catering the entire population of at least 4 out 6 provinces and regions and will serve as the safety net for the poor, enabling them to access quality care and saving them from catastrophic expenditure on health, as it provides reasonable protection of up to PKRs1 million per year for each household. Premiums are fully subsidized by the government and an insurance company manages the healthcare expenditures as a third-party administrator. The initial results of this social health protection seem encouraging. Yet, the health status variance between the different socioeconomic population groups and geographical areas is immense. Poor health conditions, limited access to primary education, safe drinking water, sanitation and hygiene conditions are some of the challenges being faced by the underprivileged population groups with per capita income of less than USD2 per day [23].

### Pakistan’s potential to achieve the UHC

For improving the state of public sector health service delivery, the district health system must be strengthened and empowered, administratively as well as financially [24]. This has been an important lever for improving key public health outcomes in low-income settings. UHC requires a new leadership agenda for public health action, through the creation of an effective mechanism of training and supportive supervision in the district health system. This new leadership paradigm should create the necessary cohesion between the different levels of the health system and evolve new linkages with other sectors, thus forging the necessary inter-sectoral collaboration and coordination at all operational levels, including meaningful participation of the community-the ultimate beneficiary of the health system [25, 26]. Improvement in access to essential health care necessitate establishing 24/7 basic health care units at least for deliveries by skilled birth attendants and strengthening the existing rural health centres, which should be catering to more than 65% of the rural inhabitants of the country. At least two provinces, Punjab and Khyber Pakhtunkhwa have started implementing action plans to revamp the primary health care system. Gaps in the human resource for health can be addressed to some extent through task shifting and expansion of the lady health workers and community midwives network in the uncovered areas of Pakistan. Community-based health workers in Pakistan, which became a global case study and known as the Lady Health Workers (LHWs) Program, has been an exemplary initiative to reach out to the remote and inaccessible pocket of population [27]. LHWs have been instrumental in providing the essential and basic maternal, newborn and chid health care in households to women, especially those who otherwise could not travel to a health facility due to a conservative cultural milieu. Another innovative model could be posting physicians to rural areas to serve primary health care centres on rotation and not on a permanent basis, with monetary incentives and by making these rotations mandatory for the post-graduation exams. The strategic direction of health sector to be adopted should bear in mind the intention of Pakistan to move towards UHC, as this requirement subsumes many of the requirements and capacities needed for International Health Regulations implementation as well [28]. The need for qualified human resources i.e., doctors, nurses, epidemiologists, etc. would have to be fulfilled to implement the proposed plans. Hence, the provinces and regions will have to invest in these capacities.

Political economy and health financing reforms are the proclaimed major drivers for achieving UHC [29]. Government of Pakistan’s landmark social protection for health initiative *“Sehat Sahulat Program”* is safeguarding the poor segments of the country’s population from the catastrophic expenditure. This program must be sustained by providing a legal cover through parliamentary legislation and must be scale up to entire country. Contrary to the present design of the program, the health system should move towards the implementation of progressive income-rated contributions to health financing, with a focus on need-based entitlements to health services, and pursuit of the concept of income and risk cross-subsidization, whereby the rich cross-subsidize the poor, whilst the healthy cross-subsidize the sick. This approach will ensure the element of equity and fairness in health [30]. Moreover, it is important to allocate the necessary resources to the health sector to fulfill the mandate of effective service delivery. The reform of public health financing should be focused on the district health system, instituting output-based budgeting and promoting performance-based budgeting [31]. However, such financial reforms will require building the management capacity at the district level, by introducing financial decentralization, and giving more autonomy to the hospitals to retain and use their revenue to improve the quality of services [32]. Without a competent and skilled management cadre that has the capacity to manage funds, merely more money allocation either through government or donor finance, would not help in achieving universal health coverage.

In spite of the fact that Pakistan’s political leadership is geared up to achieve the ambitious UHC agenda, there are a few pre-requisites that need to be fulfilled:

- Budgetary allocation for health as a percentage of GDP must be increased
- Health system’s readiness especially in the public sector ought to improve in terms of human resources and the availability of essential services
- Safety nets for health must continue regardless of the change in the political regimes
- Decrease the reliance on donors’ funding
- Accountability to be ensured across the board for service providers, managers, administrators and policymakers in the health system.

### Way forward

Providing affordable health care to the entire population is the key national agenda of the Government of Pakistan [33]. However, a large proportion of the population in Pakistan is not yet covered, and hence lacks the necessary financial protection while accessing health services. There is no denial of the centrality of social health protection scheme in achieving UHC, however, optimal utilization of health services will happen only by ensuring adequate human resources and quality health care. It is obvious and evident from other countries’ examples in the region such as Thailand and the Philippines that the success of UHC will induce a significant and lasting impact on the population’s health [34, 35]. It is to be noted that social health protection and UHC contribute not only to health; but also to poverty reduction and to the advancement of many SDGs that are linked to health [36].

However, it is also a fact that Pakistan’s per capita health expenditure is far below the cost estimates projected for accessing basic health services [37]. The government is therefore mandated to explore innovative strategies enabling the country to protect its vulnerable population against the financial risks of ill health, by raising the efficiency of its health services, while striving to mobilize the additional resources necessary, just in line with the global UHC agenda [38]. Government of Pakistan has recognized health as a public good and has pledged that no one should fall into poverty because of out of pocket healthcare expenses [39]. To attain a decent level of efficiency and effectiveness, it will be necessary to regularly review the UHC priority benefit package and periodically raising the following questions:

- Which health challenges and problems entail significant burden of disease?
- Which among these could be addressed most cost-effectively; are affordable and contribute to social justice and equity, while producing improvements in the health status of the millions of vulnerable population in Pakistan?
- Which of these interventions can generate active community participation and offer high service utilization potential?
- Which of the envisaged programmatic interventions could create high-level national and international partnerships?

The priority-setting process will need to be aligned with the SDGs to ensure that the agenda for action towards 2030 is comprehensively addressed and successfully accomplished preferably before but hopefully not beyond the targeted dates. Government of Pakistan has announced that it will implement the UHC reforms agenda on a fast track basis once the pandemic is under control. It is needless to say that in present times it is not only the virus which was killing people, it’s the poverty, lack of access and years and years of living with health conditions and health systems that have not been properly managed. The health disparities have widened because of socio-economic class and many other factors which pushed people below the poverty line. More specifically, the out of pocket expenditure on health has led to an impoverishment of scores of families because of catastrophic expenditures incurred on hospitalization during the COVID-19 times [40].

## Conclusion

Although basic health should be seen as a fundamental human right, however in the present health system, it is unfortunately determined by how much money one has and the ability to pay. COVID-19 pandemic has uncovered and ripped away the bandages from a really old wound of not only developing but many developed countries i.e., failure to deliver equitable healthcare and to acknowledge that health security is not a non-figurative concept, it is about fairness and equity. Hope, Pakistan will soon come out of this pandemic crisis, safeguarding its poor segments of the population by expanding its safety net for health, and thus achieving the UHC agenda by 2030.

## Data Availability

Not applicable.

## Abbreviations

COVID-19: Corona Virus Disease-2019
DHIS: District Health Information System
GDP: Gross Domestic Product
OOP: Out of Pocket
PDHS: Pakistan Demographic & Health Survey
SDGs: Sustainable Development Goals
UHC: Universal Health Coverage
WHO: World Health Organization
